# Supplementary material for: Phase-dependent EEG decoding of continuous visual stimuli
Source: iScience. 2026 Oct 15;29(11):117870. doi: 10.1016/j.isci.2026.117870 (PMC13694887; doi:10.1016/j.isci.2026.117870)
Supplement: Supplementary file 1 [file mmc1.pdf]

iScience, Volume *29*

Supplemental information

Phase-dependent EEG decoding of continuous visual stimuli

Michele Deodato and David Melcher

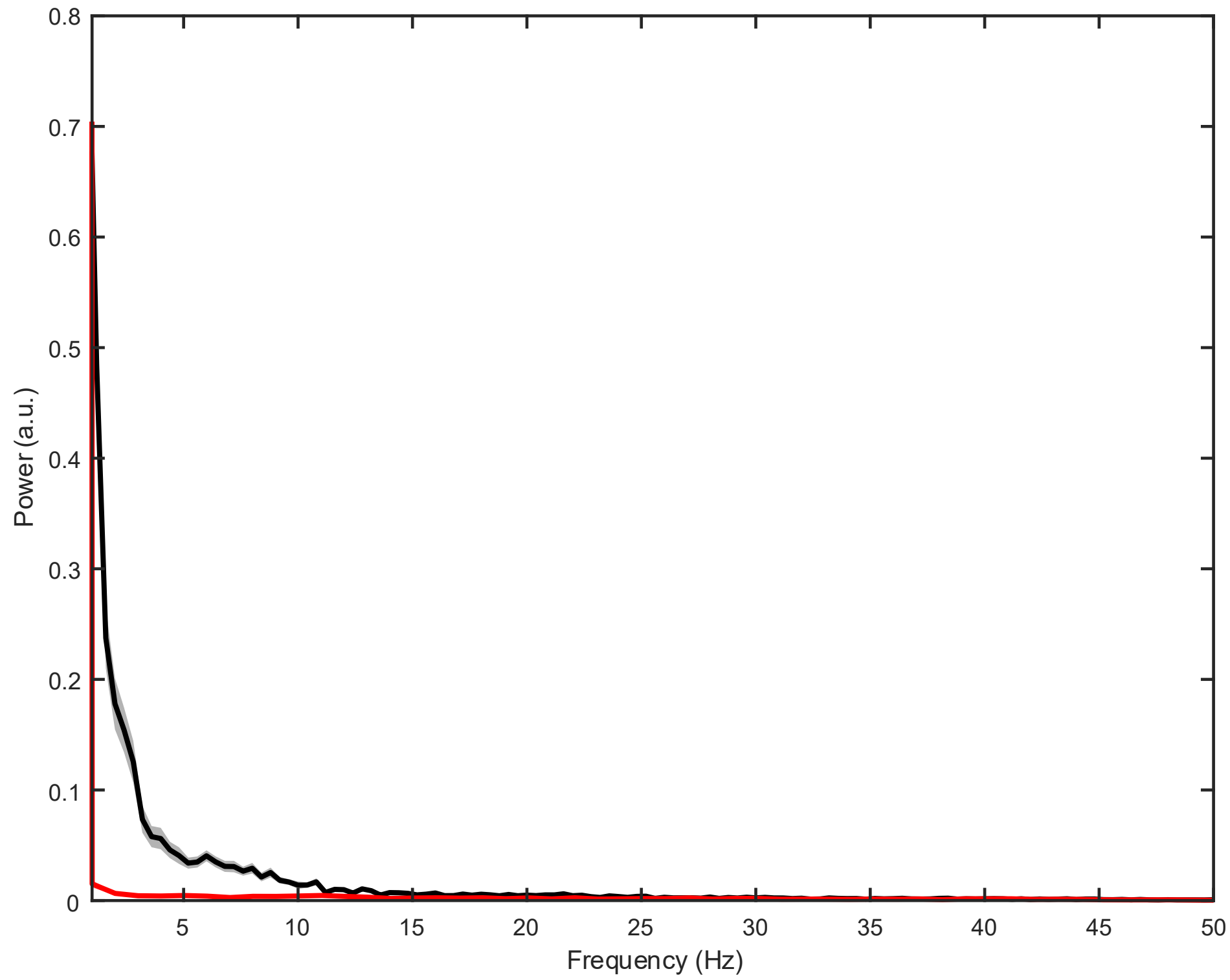

Power (a.u.)
Frequency (Hz)

1

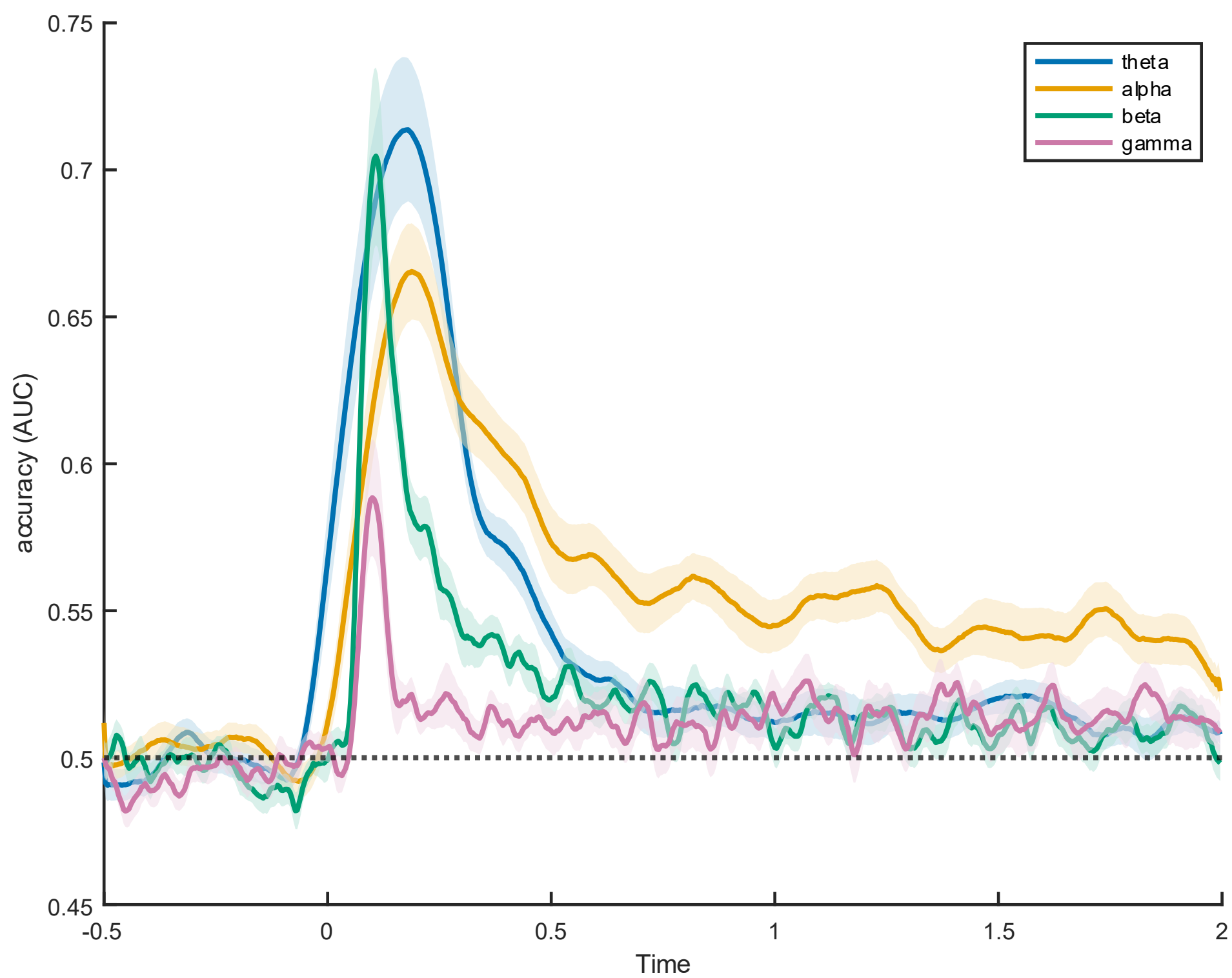

theta
alpha
beta
gamma
accuracy (AUC)
Time

2

Figure S1. (Top) Power spectrum of the full decoding time-series (black line) and only the maintenance window (1000-2000 ms; red line) averaged across participants (shaded areas indicate the standard error). Both spectra show no evidence of oscillatory peaks, suggesting that the phase-dependent decoding effect is driven by spontaneous rather than onset-locked oscillations. (Bottom) Temporal decoding of spatial frequency as a function of power in the theta (4-8 Hz), alpha (8-13 Hz), beta (14-30 Hz) and gamma (30-45 Hz) range. In the 1-2s 'maintenance' window, accuracy was strongest for alpha power but above chance for all frequency bands. Shaded areas indicate the standard error of the mean.

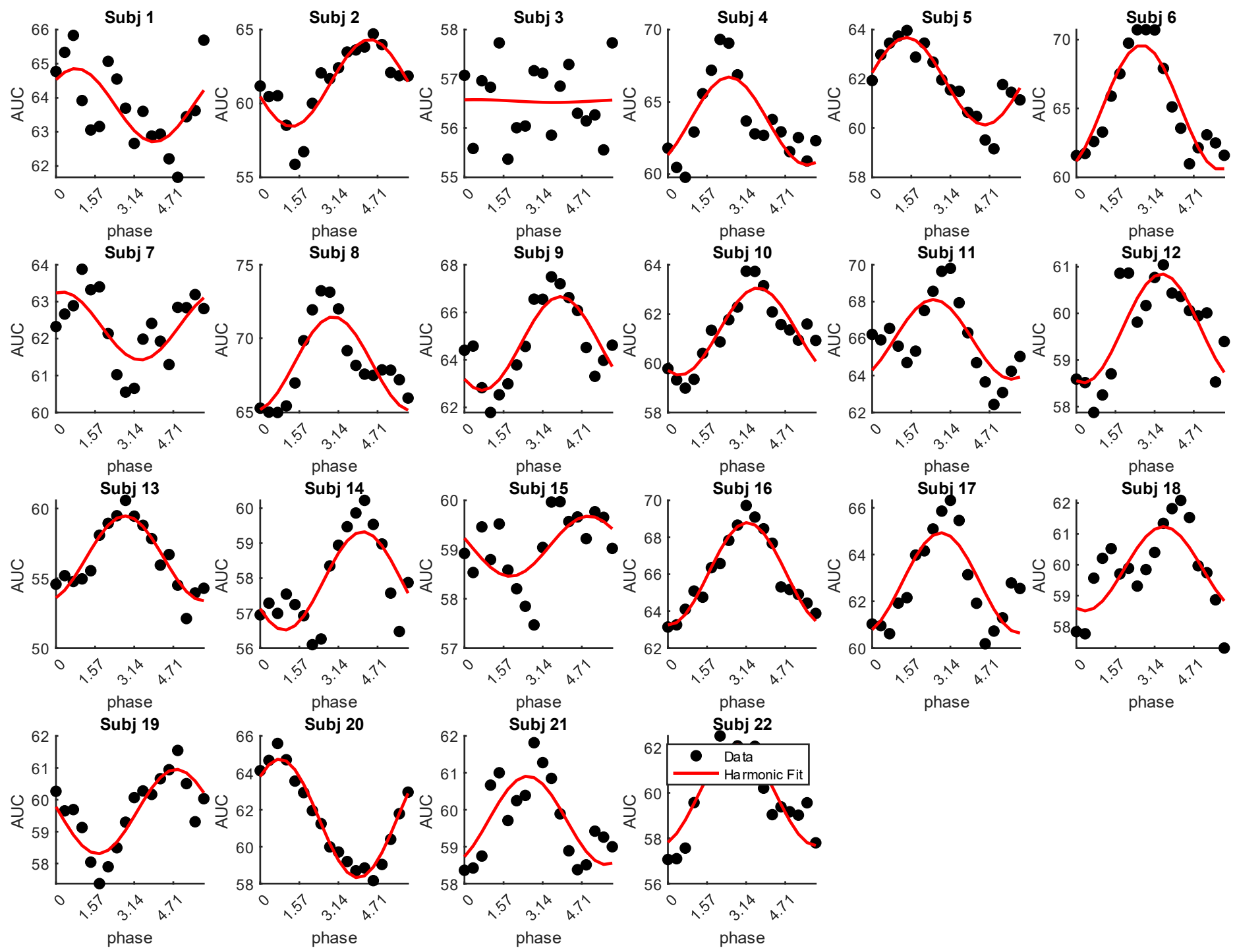

Figure S2. Individual tuning curves of spatial frequency decoding with respect to alpha phase at channel POz. Individual differences in preferred phase result in smaller average group effects.

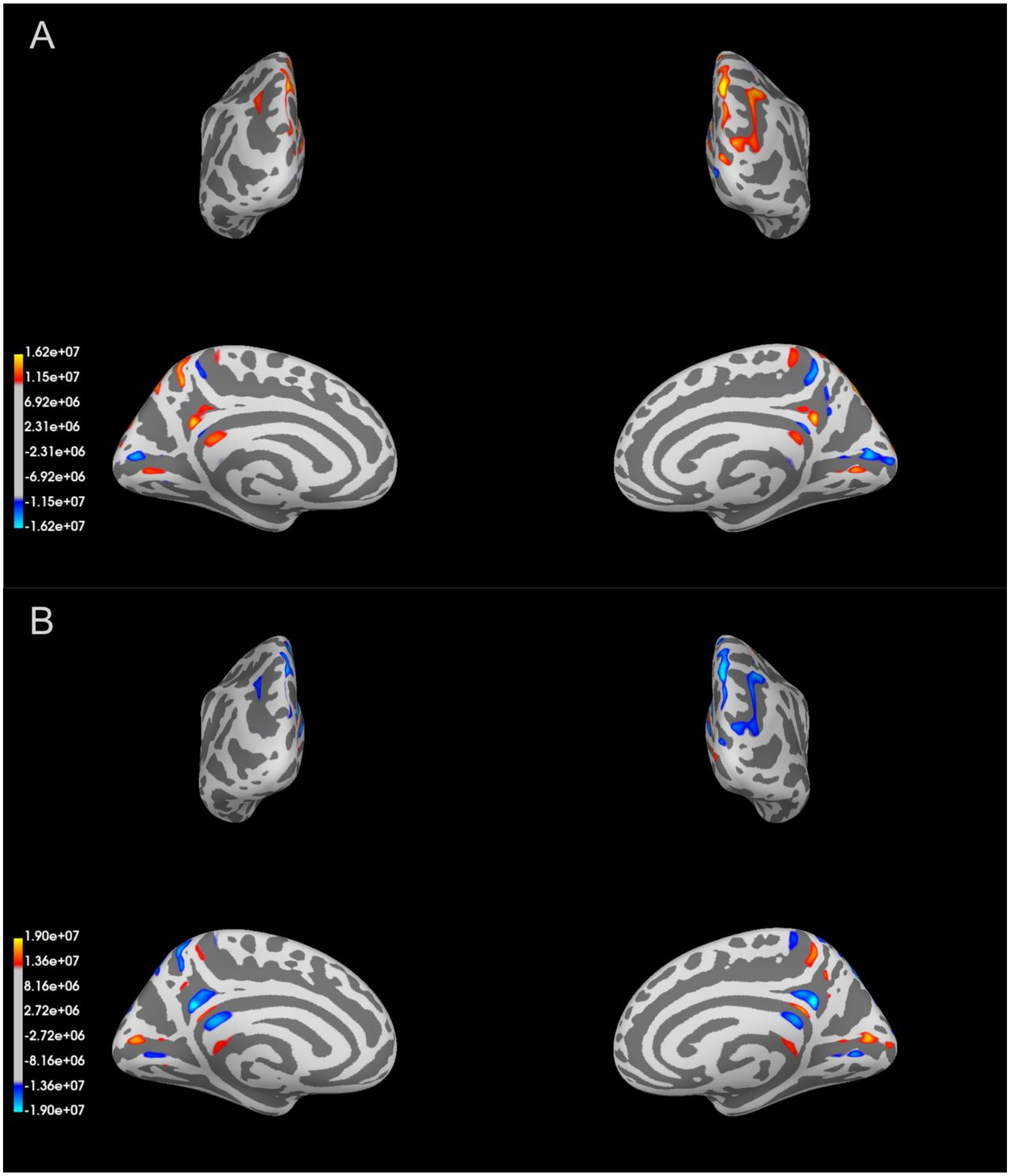

Figure S3. Template-based source reconstruction of EEG voltage topographies at the peak and trough of the alpha rhythm. Instantaneous alpha phase was defined at channel POz, and scalp voltage topographies were averaged across observations within the peak (A) and trough (B) phase bins for each participant (see methods). Because individual anatomical MRIs and digitized electrode positions were unavailable, forward solutions were constructed using the fsaverage anatomy, standard BrainVision electrode coordinates, a three-layer boundary-element model, and an ico-5 cortical source space. Signed surface-normal source estimates were obtained using sLORETA with loose orientation constraint = 0.2, depth weighting = 0.8, and $\lambda^2 = 1/9$ (assumed SNR = 3), and were subsequently averaged across participants in the common fsaverage source space. The similar spatial distributions with reversed polarity are consistent with the recruitment of a common set of occipital-parietal generators at opposite phases of the alpha cycle. Given the use

of template anatomy and standard electrode positions, localization should be interpreted at a broad regional level.
